# A magic bullet to specifically eliminate mutated mitochondrial genomes from patients' cells

**DOI:** 10.1002/emmm.201303769

**Published:** 2014-03-12

**Authors:** Carlos T Moraes

**Affiliations:** Neurology and Cell Biology, University of Miami Miller School of MedicineMiami, FL, USA

## Abstract

When mitochondrial diseases result from mutations found in the mitochondrial DNA, engineered mitochondrial-targeted nucleases such as mitochondrial-targeted zinc finger nucleases are shown to specifically eliminate the mutated molecules, leaving the wild-type mitochondrial DNA intact to replicate and restore normal copy number. In this issue, Gammage and colleagues successfully apply this improved technology on patients' cells with two types of genetic alterations responsible for neuropathy ataxia and retinitis pigmentosa (NARP) syndrome and Kearns Sayre syndrome and progressive external ophthalmoplegia (PEO).

Mitochondrial diseases are very heterogeneous, both clinically and genetically. Central nervous system and muscle are commonly affected due to their high-energy demands (Schon *et al*, 2012). Many of these disorders are caused by mutations in nuclear genes and follow a Mendelian pattern of inheritance and expression. Others are caused by mutations in the mitochondrial DNA (mtDNA), which behave differently in many aspects. Besides being exclusively maternally inherited or sporadic, deleterious mutations are usually present in a heteroplasmic condition. This happens because a cell contains approximately 1000 copies of mtDNA molecules, and mutated mtDNAs can co-exist with wild-type ones. The balance between mutated and wild-type mtDNA has a major impact on the development of a disease, as more than 80% mutated mtDNA is usually required for a biochemical and clinical phenotype (Schon *et al*, 2012).

Because of this unique aspect of mitochondrial diseases, the ability to reduce the levels of mutated mtDNA has been the goal of various laboratories. In this issue of *EMBO Molecular Medicine*, Gammage and colleagues describe the use of mitochondrial-targeted zinc finger nucleases (mtZFN) to alter the mitochondrial DNA (mtDNA) heteroplasmy balance in cells derived from patients with mitochondrial diseases (Gammage *et al*, 2014). ZFN are modular proteins that can be engineered to recognize new DNA sequences. They also contain a *Fok*I moiety at the C-terminus that cleaves the DNA, adjacent to the zinc finger-binding site. Because the *Fok*I nuclease moiety is functional as a dimer, for each region to be cleaved, two juxtaposed DNA recognition/nuclease monomers are required to promote site-specific double-strand breaks (Palpant & Dudzinski, 2013).

Gammage and colleagues developed two ZFN that can bind specific regions of the mtDNA. The authors had to modify the ZFN so that the protein was localized to the mitochondrial matrix (mtZFN), where they can physically interact with the mtDNA. To increase specificity of DNA cleavage, they used an obligatory heterodimeric form of *Fok*I. They tested the approach with two different mutations, a point mutation associated with a neurological disease known as neuropathy ataxia and retinitis pigmentosa (NARP) syndrome and a large deletion breakpoint associated with disorders known as Kearns Sayre syndrome and progressive external ophthalmoplegia. In both cases, expression of the specific mtZFN molecular pairs led to a decrease in the relative levels of mutated mtDNA. These results are similar to those recently obtained for a different class of designer nucleases, known as mitoTALEN (Bacman *et al*, 2013).

expression of the specific mtZFN molecular pairs led to a decrease in the relative levels of mutated mtDNA.

The ability to reduce the levels of mutated mtDNA by designer mitochondrial nucleases can have a major effect on the biochemical phenotype of affected cells. A small decrease in the levels of mutated mtDNA would induce the residual mtDNA to proliferate and repopulate the mitochondria with a higher ratio of wild-type to mutated mtDNA (Fig 1). mtDNA levels are controlled by a poorly understood mechanism, but likely by factors responsible for mtDNA replication, such as transcription factors (required for replication priming), helicase twinkle, DNA polymerase gamma, and others (Carling *et al*, 2011). Because the wild-type mtDNA is very protective in heteroplasmic cells, a decrease in the percentage of mutant from 80% to 60% can completely eliminate the biochemical defect (Sciacco *et al*, 1994). Indeed, Gammage and colleagues showed an improvement in mitochondrial respiration function in cells harboring high levels of a deletion mutant mtDNA after treating them with a mtZFN specific for the mutated molecules (Gammage *et al*, 2014).

**Figure 1:**
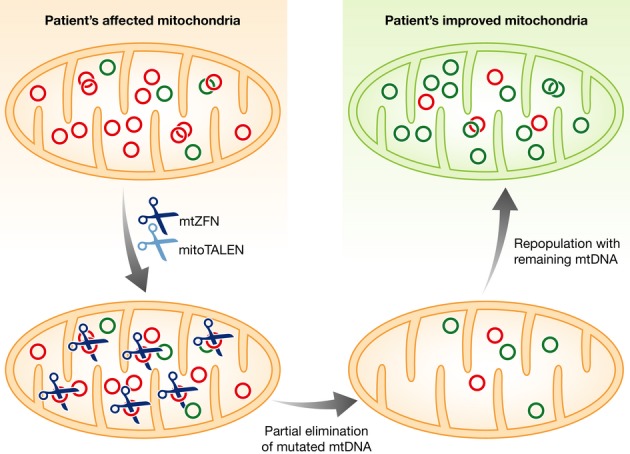
Mitochondrial-targeted nucleases such as mtZFN (Gammage *et al*, 2014) or mitoTALEN (Bacman *et al*, 2013) can specifically eliminate mutated mtDNA molecules, leaving the wild-type mtDNA intact. After a double-strand break, most molecules are rapidly degraded. The residual mtDNA is stimulated to replicate and restore the normal mtDNA copy number. The final result is a cell with higher levels of wild-type mtDNA and an improved biochemical phenotype, as shown by Gammage and colleagues in this issue (Gammage *et al*, 2014).

after decades of cold leads, this approach offers a first concrete glimpse of hope for the treatment of these devastating metabolic disorders.

What are the next steps toward translating this exciting technology to clinical use? How can we move it from cultured cells to whole organs? As in any gene therapy goal, delivery of the desired gene or gene product to affected tissues remains a challenge. This is particularly difficult for mtZFN or mitoTALEN because of their large sizes. Viral vectors, such as AAV (Mingozzi and High, 2011), are one of the tools of choice, and although they have limitations on the size of the DNA that can be packaged, mtZFN may be a bit smaller and more amenable to packaging in a single virion when compared to mitoTALEN. Another challenge associated with this approach is the control of mtDNA degradation, as a severe, potentially catastrophic depletion may ensue if the levels of mutated mtDNA are high and the nuclease is highly efficient. Animal experiments have suggested that skeletal muscle and heart can tolerate temporary degradation of mtDNA by restriction endonucleases (Bacman *et al*, 2010, 2012). However, it is possible that some tissues (e.g., skeletal muscle) can tolerate a transient reduction in total mtDNA levels, before repopulation by the wild-type mtDNA, better than others (e.g., CNS).

Despite future challenges, after decades of cold leads, this approach offers a first concrete glimpse of hope for the treatment of these devastating metabolic disorders.
